# Digital Meditation to Target Employee Stress: A Randomized Clinical Trial

**DOI:** 10.1001/jamanetworkopen.2024.54435

**Published:** 2025-01-14

**Authors:** Rachel M. Radin, Julie Vacarro, Elena Fromer, Sarah E. Ahmadi, Joanna Y. Guan, Sarah M. Fisher, Sarah D. Pressman, John F. Hunter, Kate Sweeny, A. Janet Tomiyama, Lauren Tiongco Hofschneider, Matthew J. Zawadzki, Larisa Gavrilova, Elissa S. Epel, Aric A. Prather

**Affiliations:** 1 Department of Psychiatry and Behavioral Sciences, University of California San Francisco; 2 Department of Psychological Sciences, University of Connecticut, Storrs; 3 Department of Psychology, University of California, Davis; 4 Department of Psychological Science, University of California, Irvine; 5 Department of Psychology, University of California, Riverside; 6 Department of Psychology, University of California, Los Angeles; 7 Department of Psychological Sciences, University of California, Merced

## Abstract

**Importance:**

Mindfulness meditation may improve well-being among employees; however, effects of digital meditation programs are poorly understood.

**Objective:**

To evaluate the effects of digital meditation vs a waiting list condition on general and work-specific stress and whether greater engagement in the intervention moderates these effects.

**Design, Setting, and Participants:**

This randomized clinical trial included a volunteer sample of adults (aged ≥18 years) employed at a large academic medical center who reported mild to moderate stress, had regular access to a web-connected device, and were fluent in English. Exclusion criteria included being a regular meditator. Participants were recruited from May 16, 2018, through September 28, 2019, and completed baseline, 8-week, and 4-month measures assessing stress, job strain, burnout, work engagement, mindfulness, depression, and anxiety. Data were analyzed from March 2023 to October 2024.

**Intervention:**

Participants were randomized 1:1 to a digital meditation program or the waiting list control condition. Participants in the intervention group were instructed to complete 10 minutes of meditation per day for 8 weeks. The control group was instructed to continue their normal activities and not add any meditation during the study period.

**Main Outcomes and Measures:**

The primary outcome measure was change in Perceived Stress Scale (PSS) score at 8 weeks. Secondary outcome measures included changes in job strain, measured as work effort-reward imbalance.

**Results:**

A total of 1458 participants (mean [SD] age, 35.54 [10.30] years; 1178 [80.80%] female) were included. Those randomized to meditation (n = 728) vs waiting list (n = 730) showed improvements in PSS (Cohen *d*, 0.85; 95% CI, 0.73-0.96) and in all secondary outcome measures (eg, job strain: Cohen *d*, 0.34; 95% CI, 0.23-0.46) at 8 weeks. These improvements were maintained at 4 months after randomization (PSS: Cohen *d*, 0.71; 95% CI, 0.59-0.84; job strain: Cohen *d*, 0.37; 95% CI, 0.25-0.50). Those using the app from 5 to 9.9 min/d vs less than 5 min/d showed greater reduction in stress (mean PSS score difference, −6.58; 95% CI, −7.44 to −5.73).

**Conclusions and Relevance:**

The findings suggest that a brief, digital mindfulness-based program is an easily accessible and scalable method for reducing perceptions of stress. Future work should seek to clarify mechanisms by which such interventions contribute to improvements in work-specific well-being.

**Trial Registration:**

ClinicalTrials.gov Identifier: NCT03527303

## Key Points

**Question:**

Can digital mindfulness meditation improve general stress and work-related stress among employees at a large academic medical center?

**Findings:**

In this randomized clinical trial of 1458 employees, those who received mindfulness meditation (vs waiting list control) had significant reductions in perceived stress at 8 weeks.

**Meaning:**

The findings suggest that participating in a brief digital mindfulness-based program is an effective method for reducing general and work-related stress in employees.

## Teaser

This randomized clinical trial investigates whether a digital mindfulness meditation application reduces perceptions of global and job-related stress among adults employed at a large academic medical center.

## Introduction

Mental health is at an historic low in the US,^1,2^ and work stress may be a primary contributor.^3,4^ Work stress is associated with poorer emotional and physical well-being^5,6^ as well as high absenteeism and low presenteeism.^7^ Around 8% of US health care costs are attributable to work-related stressors.^4^ The COVID-19 pandemic has worsened these impacts,^8^ particularly among health care professionals,^3,4^ with 45% reporting high levels of job burnout.^1^

Mindfulness meditation may reduce work-related stress,^9,10^ as it aims to cultivate a nonjudging awareness of the present moment and promote self-regulation.^11^ Workplaces have invested in in-person mindfulness programs; however, these formats cannot be easily scaled and disseminated, making them less cost-effective and leaving many without adequate opportunities for reducing stress. Mindfulness delivered via self-guided smartphone apps may offer convenient alternatives,^12,13^ with the benefit of standardization of instruction, and participants can control how they access treatment.

Mindfulness apps are associated with significant reductions in perceived stress and improvements in well-being, with small to medium effect estimates maintained at 6-month follow-up.^14,15,16^ These outcomes have not been studied extensively in the workplace, although there may be small, positive changes in well-being and work effectiveness.^17^ In a small sample randomized to digital mindfulness (vs waiting list), our team found significant, sustained improvements in well-being, job strain, and blood pressure, particularly for participants who meditated more.^18^ Many existing studies are limited by an inability to determine treatment adherence.

In the current study, we randomized a large sample of employees at an academic medical center to a commercially available, digitally delivered meditation platform or a waiting list control condition. We investigated immediate treatment effects and maintenance of improvements in global perceptions of psychological distress and, secondarily, work stress, job strain, burnout, work engagement, subjective mindfulness, and symptoms of depression and anxiety. We also examined the influence of treatment adherence on outcomes. We hypothesized that participants randomized to the meditation condition would outperform those randomized to the waiting list control condition with respect to improvements in all primary and secondary outcomes and that treatment adherence would moderate these effects.

## Methods

### Participants

This randomized clinical trial (NCT03527303) was approved by the University of California San Francisco (UCSF) institutional review board, and all participants provided electronic informed consent. The trial protocol and statistical analysis plan are given in Supplement 1. We followed the Consolidated Standards of Reporting Trials (CONSORT) reporting guideline for all data outcomes.

Eligible participants were aged 18 years or older, were employed within the same UCSF health system, reported mild to moderate levels of stress in the previous month (Perceived Stress Scale [PSS]^19^ score of ≥15 on a scale of 0-40, with higher values indicating more stress), had regular access to a web-enabled device, and were fluent in English. Exclusion criteria included being an experienced meditator (having a sitting meditation practice ≥3 times/wk in the past 3 months). Participants self-reported race and ethnicity from the National Institutes of Health Office of Management and Budget revised 5 minimum categories for race: American Indian or Alaska Native, Asian, Black or African American, Native Hawaiian or Pacific Islander, White, and other or unknown (multiracial, not listed, and no response). Ethnicity categories were Hispanic or Latino and not Hispanic or Latino. Race and ethnicity were collected to provide general descriptive data of study participants.

Our group’s prior study^18^ detected effects of a digital mindfulness intervention among fewer than 250 participants. For the current study, we enrolled as many employees as feasible, yielding 1458 participants. Participants did not receive monetary compensation but received a 1-year subscription to an app for mindfulness meditation (valued at $150) and were entered into a raffle (prizes valued at $1000). We enrolled participants from May 16, 2018, through September 28, 2019. Individuals were recruited through passive recruitment (eg, flyers) and in-person methods (eg, tables at staff events).

### Study Design

Participants completed baseline self-report assessments and were randomized to 1 of 2 conditions: digital meditation (MED) or a waiting list control (WL). We reassessed participants on all measures at 8 weeks and on some measures at 4 months.

### Randomization and Procedures

The randomization sequence was generated ahead of time by an online randomization generator preprogrammed for 1:1 randomization in Qualtrics. The sequence was concealed until analysis; however, research assistants (including J.V.) were unblinded to track adherence and provided technical assistance. Randomization was not stratified by any participant characteristics and was triggered on completion of baseline assessments. Interested individuals completed an online screener and, if eligible, were provided a consent form.

### Interventions

#### MED Condition

Participants in MED were provided a subscription code and installation instructions for Headspace (Headspace, Inc), an app that provides guided and unguided mindfulness meditations. We instructed participants to complete 10 minutes of daily meditation for 8 weeks starting with the Basics course (30 sessions focused on learning fundamentals of meditation and mindfulness, deepening the practice, and applying mindfulness to everyday life), followed by the Letting Go of Stress course (30 sessions focused on developing awareness of stress and learning to reframe negative emotions), although they had access to use any course they wished. We telephoned, emailed, and texted participants who completed less than 1 meditation in the first 2 weeks of their intervention period to troubleshoot technical problems and increase engagement.

#### WL Control Condition

We instructed WL participants to continue normal activities and avoid initiating a meditation practice during the study period. We provided these participants with a 1-year subscription to the study app after completion of 4-month questionnaires.

### Outcomes

#### Primary Outcome Measure

Perceived stress at 8 weeks after randomization was the primary outcome for this study, measured using the 10-item PSS,^19^ which assesses a person’s evaluation of the life stress they have experienced over the previous month. The PSS has demonstrated adequate reliability and validity in similar populations.^20^ We observed high reliability (Cronbach α = 0.85-0.89).

#### Secondary Outcome Measures

##### Job Strain, Job Overcommitment, and Burnout

Job strain was assessed using the 22-item Siegrist Effort-Reward Imbalance Scale.^21^ Participants rated statements that reflect perceived responsibility and demands of their job (ie, effort) as well as perceived respect and support (ie, reward), with responses scored on a range from 1 to 4 and higher scores indicating greater strain. This measure yields an effort subscale (5 items; score range, 5-25, with higher scores indicating greater effort) and a reward subscale (11 items; score range, 11-55, with higher scores indicating more reward). Total score was calculated as the ratio of demand to reward, with higher ratios reflecting greater job strain. This measure also yields an overcommitment subscale (6 items), which reflects the tendency toward overengagement and becoming overwhelmed by one’s job. The subscale provides a score range from 6 to 24, with higher scores reflecting more overcommitment. Scale reliability was high (Cronbach α = 0.81).

Burnout was measured using the Bergen Burnout Inventory (BBI), a well-validated 9-item measure.^22^ The BBI includes 3 subscales that assess feelings of exhaustion, cynicism, and inadequacy at work. A mean total score (range, 1-6) was calculated, with higher scores reflecting greater burnout. Scale reliability was high (Cronbach α = 0.84).

##### Work Engagement

Work engagement was measured using the Utrecht Work Engagement Scale (UWES),^23^ a 9-item scale that assesses how immersed and content an employee is in their work. A mean total score was computed (range, 1-6), with higher scores indicating greater engagement. There are 3 subscales: vigor, dedication, and absorption. Scale reliability was high (Cronbach α = 0.89).

##### Subjective Mindfulness

Mindfulness was assessed using the extensively validated Mindfulness Attention Awareness Scale (MAAS), a 15-item scale that assesses dispositional mindfulness.^11^ A mean total score was computed (range, 1-6), with higher scores reflecting greater mindfulness. Scale reliability was high (Cronbach α = 0.89).

##### Depressive and Anxious Symptoms

We measured depressive symptoms using the 8-item Patient Health Questionnaire (PHQ-8)^24^ (range, 0-24, with higher scores indicating greater symptom severity). Scale reliability was high (Cronbach α = 0.82). We assessed anxiety symptoms using the 7-item Generalized Anxiety Disorder (GAD-7)^25^ questionnaire (range, 0-21, with higher scores indicating greater symptom severity). Scale reliability was high (Cronbach α = 0.87).

##### Treatment Adherence

Treatment adherence data were provided by the study app. We operationalized adherence as the mean number of meditation minutes per day.

### Statistical Analyses

#### Data Preparation

Data were analyzed from March 2023 to October 2024. All analyses used SPSS, version 29.0 (IBM Corp). Summary statistics were computed to evaluate the distributions of primary and secondary measures and assess outliers (none were detected).

#### Attrition or Dropout Analysis

We used *t* tests to compare the differences in means of baseline outcomes for participants who dropped out in the MED arm vs WL arm at each follow-up (eTable 1 in Supplement 2). Among dropouts, there was no difference between groups on any measure.

#### Treatment Effect on Outcome Variables

For all analyses, we used an intent-to-treat approach, providing a conservative estimate of treatment effects. For measures collected at all 3 time points (baseline, 8 weeks, and 4 months), we ran 4 separate linear mixed-model analyses, each including 1 of the 4 measures as the dependent variable, participant identification number as a random effect, and treatment group (independent variable: MED vs WL) as a fixed effect. All assumptions were met with regard to linearity, normality of the distribution of residuals, and random effects. We bootstrapped 1000 sample replicates from the initial model to calculate posterior estimates (eFigures 2, 4, 6, and 8 in Supplement 2).

For measures collected at 2 time points, we used *t* tests to compare mean changes in each outcome between arms (independent variable: MED vs WL). Change scores were calculated by subtracting the baseline from 8-week value. We included baseline value of the dependent variable as a covariate. In all analyses, we considered *P* ≤ .05 to be statistically significant (using 2-tailed tests), and we included 95% CIs for better interpretation of findings.

#### Exploratory Analysis of Effect of Treatment Adherence

Among participants randomized to MED, we used general linear models to examine the impact of level of treatment adherence on changes in the primary outcome. Categories were low (<5 min/d of meditation), medium (5-9.9 min/d), and high (≥10 min/d).

## Results

### Participant Recruitment and Retention

**Figure 1.**
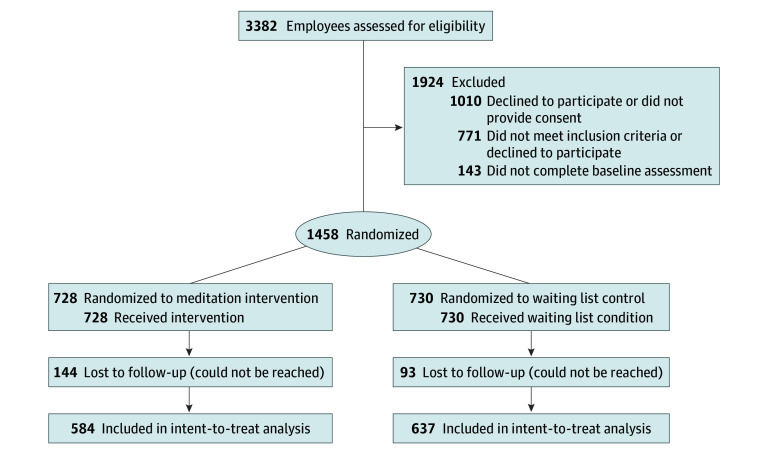
CONSORT Flow Diagram

A total of 3382 individuals completed the screener, of whom 2611 (77.20%) were deemed eligible to participate. A total of 1465 participants were randomized to 8 weeks of MED or the WL control. Seven individuals (0.48%) were randomized in error and withdrawn, resulting in 1458 participants: 728 randomized to MED and 730 randomized to the WL control (Figure 1). Attrition was relatively low overall (237 participants [16.23%]). A greater percentage of attrition occurred among those randomized to MED (144 [19.78%]) than to the WL group (93 [12.74%]).

### Participant Characteristics

Table 1 provides baseline sample characteristics. Mean (SD) participant age was 35.54 (10.3) years. Of all 1458 participants, 1178 (80.80%) identified as female and 261 (17.90%) as male. Two (0.14%) were American Indian or Alaska Native; 399 (27.37%), Asian; 50 (3.43%), Black or African American; 14 (0.96%), Native Hawaiian or Pacific Islander; 755 (51.78%), White; 79 (5.42%), multiracial; and 159 (10.90%), other race or not reported. A total of 169 (11.59%) were Hispanic or Latino, and 1289 (88.41%) were not Hispanic or Latino. The most common occupations were academic research staff (413 [28.33%]), medical staff (321 [22.02%]), and administrative staff (277 [19.00%]). By design, participants had a mean (SD) PSS score indicative of moderate stress (21.42 [4.86]),^26^ and the majority (1304 [89.44%]) reported meditating less than once a month to never.

**Table 1.** Baseline Health Characteristics

Characteristic	Participants^a^		
	Total (N = 1458)	MED group (n = 728)	WL control (n = 730)
Age, mean (SD), y	35.54 (10.30)	35.54 (10.49)	35.53 (10.12)
Gender			
Identifying as female	1178 (80.80)	603 (82.83)	575 (78.77)
Identifying as male	261 (17.90)	113 (15.52)	148 (20.27)
Other or not reported	19 (1.30)	12 (1.65)	7 (0.96)
Race			
American Indian or Alaska Native	2 (0.14)	1 (0.14)	1 (0.14)
Asian	399 (27.37)	196 (26.92)	203 (27.81)
Black or African American	50 (3.43)	30 (4.12)	20 (2.74)
Native Hawaiian or Pacific Islander	14 (0.96)	4 (0.55)	10 (1.37)
White	755 (51.78)	377 (51.79)	378 (51.78)
Multiracial	79 (5.42)	35 (4.81)	44 (6.03)
Other or not reported^b^	159 (10.90)	85 (11.68)	74 (10.14)
Ethnicity			
Hispanic or Latino	169 (11.59)	85 (11.68)	84 (11.51)
Not Hispanic or Latino	1289 (88.41)	643 (88.32)	646 (88.49)
Educational attainment			
<4-y college degree	81 (5.56)	35 (4.81)	46 (6.30)
4-y college degree	564 (38.68)	278 (38.19)	286 (39.18)
Graduate degree	813 (55.76)	415 (57.01)	398 (54.52)
Annual household income, $			
<50 000	187 (12.83)	93 (12.78)	94 (12.88)
50 000 to <100 000	470 (32.24)	222 (30.50)	248 (33.97)
100 000 to <200 000	453 (31.07)	219 (30.08)	234 (32.05)
≥200 000	285 (19.55)	155 (21.29)	130 (17.81)
Prefer not to answer or no response	63 (4.32)	39 (5.36)	24 (3.29)
Position			
Academic faculty	116 (7.96)	61 (8.38)	55 (7.53)
Administrative staff	277 (19.00)	136 (18.68)	141 (19.32)
Medical staff	321 (22.02)	162 (22.25)	159 (21.78)
Midlevel management	158 (10.84)	81 (11.13)	77 (10.55)
Researcher or research associate	413 (28.33)	200 (27.47)	213 (29.18)
Service staff	7 (0.48)	3 (0.41)	4 (0.55)
Senior leadership	7 (0.48)	4 (0.55)	3 (0.41)
Other or no response	159 (10.91)	81 (11.13)	78 (10.68)
PSS score, mean (SD)^c^	21.42 (4.86)	21.41 (5.07)	21.42 (4.63)
Job strain score, mean (SD) [n]^d^			
Effort	14.88 (2.99) [1450]	14.88 (2.99) [725]	14.90 (2.99) [725]
Reward	31.17 (4.68) [1444]	31.20 (4.88) [720]	31.16 (4.47) [724]
Effort-reward ratio	0.22 (0.06) [1438]	0.22 (0.07) [717]	0.22 (0.06) [721]
Overcommitment	16.17 (3.34) [1450]	16.15 (3.40) [722]	16.19 (3.29) [728]
Burnout, BBI score, mean (SD) [n]^e^			
Total	3.17 (0.96) [1446]	3.15 (0.99) [721]	3.19 (0.94) [725]
Exhaustion	3.30 (1.12) [1456]	3.29 (1.14) [727]	3.32 (1.09) [729]
Cynicism	3.15 (1.22) [1454]	3.12 (1.24) [725]	3.18 (1.20) [729]
Inadequacy	3.06 (1.15) [1450]	3.04 (1.18) [724]	3.09 (1.13) [726]
Work engagement, UWES score, mean (SD) [n]^f^			
Total	3.51 (0.88) [1452]	3.53 (0.88) [726]	3.48 (0.89) [726]
Vigor	2.83 (1.04) [1455]	2.88 (1.06) [728]	2.79 (1.02) [727]
Dedication	3.87 (1.09) [1455]	3.90 (1.08) [728]	3.84 (1.09) [727]
Absorption	3.83 (0.95) [1455]	3.83 (0.93) [726]	3.82 (0.97) [729]
Mindfulness, MAAS score, mean (SD) [n]^g^	3.40 (0.81) [1457]	3.41 (0.80) [722]	3.38 (0.82) [725]
Depression, PHQ-8 score, mean (SD) [n]^h^	7.80 (4.58) [1455]	7.88 (4.66) [726]	7.73 (4.50) [729]
Anxiety, GAD-7 score, mean (SD) [n]^i^	8.31 (4.78) [1454]	8.42 (4.82) [728]	8.19 (4.74) [726]
Meditation frequency at screening			
Never	1097 (75.24)	553 (75.96)	544 (74.52)
<1 Time per mo	285 (19.55)	114 (15.66)	147 (20.14)
1-3 Times per mo	31 (2.13)	13 (1.79)	18 (2.47)
1-2 Times per wk	45 (3.09)	24 (3.30)	21 (2.88)

Abbreviations: BBI, Bergen Burnout Inventory; GAD-7, 7-item Generalized Anxiety Disorder; MAAS, Mindfulness Attention Awareness Scale; MED, digital meditation intervention; PHQ-8, 8-item Patient Health Questionnaire; PSS, Perceived Stress Scale; UWES, Utrecht Work Engagement Scale; WL, waiting list.

^a^ Data are presented as number or number/total number (percentage) of participants unless otherwise indicated.

^b^ Included not listed and no response.

^c^ Score range 0 to 40, with higher values indicating more stress.

^d^ Based on the Siegrist Effort-Reward Imbalance Scale, with effort (demand) subscale score range of 5 to 25, reward subscale score range of 11 to 55, and overcommitment subscale score range of 6 to 24 (higher scores indicate greater effort, reward, and job overwhelm, respectively). The effort-reward ratio is calculated as the ratio of demand to reward, with higher ratios (range, 0-1) reflecting greater job strain.

^e^ Score range 1 to 6, with higher scores reflecting greater burnout.

^f^ Score range 1 to 6, with higher scores indicating greater engagement.

^g^ Score range 1 to 6, with higher scores reflecting greater mindfulness.

^h^ Score range 0 to 24, with higher scores indicating greater depression symptom severity.

^i^ Score range 0 to 21, with higher scores indicating greater anxiety symptom severity.

### Treatment Adherence

Participants randomized to MED (n = 728) engaged with the app a mean (SD) of 5.20 (3.88) min/d. This engagement included access to any type of session (eg, mindfulness, sleep, or eating). Participants accessed meditation-specific sessions a mean (SD) of 3.36 (3.33) min/d. Thirty-one of those in the MED arm (4.26%) were 100% adherent to instructions to meditate 10 min/d or more. Most participants required at least 1 follow-up contact to improve adherence.

### Primary Outcome

**Figure 2.**
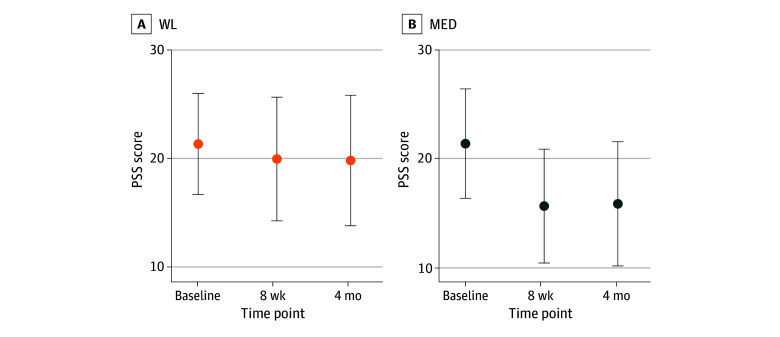
Effect of Treatment on Change in Perceived Stress Scale (PSS) Score at 8 Weeks and 4 Months Data points represent means and error bars, SEs. MED indicates digital meditation; WL, waiting list control.

There was a statistically significant effect of treatment group on changes in PSS (Cohen *d*, −0.46; 95% CI, −0.55 to −0.43; *P* < .001) (Table 2). At 8 weeks, group differences in PSS favored MED (Cohen *d*, 0.85; 95% CI, 0.73-0.96; *P* < .001); those in the MED group showed a mean (SE) reduction in PSS of −5.84 (0.22), and those in the WL group showed a mean (SE) reduction of −1.45 (0.20). At 4 months, the group differences remained (Cohen *d*, 0.71; 95% CI, 0.59-0.84; *P* < .001), with those in the MED group maintaining a greater mean (SE) reduction in PSS (−5.64 [0.27] vs −1.63 [0.22] in the WL group) (Figure 2, Table 3, and eFigure 1 in Supplement 1). In an exploratory analysis, we found that increases in 8-week MAAS score explained 23% of the effect of MED (vs WL) on reductions in PSS at 4 months.

**Table 2.** Intention-to-Treat Linear Mixed-Model Analyses of Changes in Primary and Secondary Outcomes

Measure	Estimate, β (95% CI)	*P* value	Cohen *d* (95% CI)
**Primary outcome**			
Perceived Stress Scale score (3753 observations)			
Intercept	20.29 (12.98 to 22.60)	<.001	NA
Treatment	−2.55 (−2.91 to −2.20)	<.001	−0.46 (−0.55 to −0.43)
**Secondary outcome**			
Job strain, effort-reward ratio (3683 observations)			
Intercept	0.22 (0.22 to 0.23)	<.001	NA
Treatment	−0.01 (−0.01 to −0.00)	<.001	−0.15 (−0.17 to −0.12)
Work engagement, total UWES score (3701 observations)			
Intercept	3.48 (3.41 to 3.55)	<.001	NA
Treatment	0.18 (0.12 to 0.24)	<.001	0.20 (0.17 to 0.23)
Mindfulness, MAAS score (3685 observations)			
Intercept	3.35 (3.24 to 3.45)	<.001	NA
Treatment	0.31 (0.25 to 0.36)	<.001	0.38 (0.35 to 0.41)

Abbreviations: MAAS, Mindfulness Attention Awareness Scale; NA, not applicable; UWES, Utrecht Work Engagement Scale.

**Table 3.** Intention-to-Treat Analyses of Changes in Primary and Secondary Outcomes

Measure	Participants, No.		Change, mean (SE)		Mean difference (95% CI)	*t*	*P* value^a^	Cohen *d* (95% CI)
	MED	WL	MED	WL				
Perceived Stress Scale								
8 wk	580	634	−5.84 (0.22)	−1.45 (0.20)	−4.39 (−4.97 to −3.80)	−14.70	<.001	0.85 (0.73 to 0.96)
4 mo	489	585	−5.64 (0.27)	−1.63 (0.22)	−4.02 (−4.70 to −3.33)	−11.53	<.001	0.71 (0.59 to 0.84)
Job strain								
Effort								
8 wk	578	625	−0.85 (0.09)	−0.27 (0.09)	−0.58 (−0.84 to −0.32)	−4.40	<.001	0.25 (0.14 to 0.37)
4 mo	475	571	−0.81 (0.12)	−0.26 (0.10)	−0.55 (−0.86 to −0.25)	−3.56	<.001	0.22 (0.10 to 0.34)
Reward								
8 wk	567	625	0.79 (0.15)	−0.28 (0.13)	1.07 (0.68 to 1.46)	5.36	<.001	0.31 (0.20 to 0.43)
4 mo	473	571	0.72 (0.18)	−0.55 (0.15)	1.27 (0.81 to 1.72)	5.47	<.001	0.34 (0.22 to 0.46)
Effort-reward ratio								
8 wk	565	618	−0.02 (0.00)	0.00 (0.00)	−0.02 (−0.02 to −0.01)	−5.91	<.001	0.34 (0.23 to 0.46)
4 mo	467	565	−0.02 (0.00)	0.00 (0.00)	−0.02 (−0.03 to −0.01)	−5.96	<.001	0.37 (0.25 to 0.50)
Overcommitment								
8 wk	572	629	−1.88 (0.12)	−0.71 (0.10)	−1.17 (−1.47 to −0.87)	−7.60	<.001	0.44 (0.33 to 0.56)
4 mo	474	574	−2.00 (0.13)	−0.57 (0.11)	−1.43 (−1.76 to −1.09)	−8.37	<.001	0.52 (0.40 to 0.64)
Work engagement								
Total score								
8 wk	569	619	0.20 (0.03)	0.01 (0.03)	0.19 (0.12 to 0.26)	5.38	<.001	0.31 (0.20 to 0.43)
4 mo	476	566	0.17 (0.03)	−0.06 (0.03)	0.23 (0.15 to 0.31)	5.70	<.001	0.35 (0.23 to 0.48)
Vigor								
8 wk	572	623	0.32 (0.03)	0.03 (0.03)	0.29 (0.20 to 0.38)	6.23	<.001	0.36 (0.25 to 0.48)
4 mo	479	569	0.29 (0.04)	0.00 (0.04)	0.29 (0.19 to 0.39)	5.49	<.001	0.34 (0.22 to 0.46)
Dedication								
8 wk	573	623	0.12 (0.03)	−0.03 (0.03)	0.16 (0.08 to 0.24)	3.70	<.001	0.21 (0.10 to 0.33)
4 mo	479	569	0.12 (0.04)	−0.12 (0.04)	0.24 (0.14 to 0.34)	4.63	<.001	0.29 (0.17 to 0.41)
Absorption								
8 wk	570	623	0.16 (0.03)	0.03 (0.03)	0.13 (0.04 to 0.22)	2.95	.003	0.17 (0.06 to 0.29)
4 mo	478	573	0.10 (0.03)	−0.08 (0.03)	0.18 (0.09 to 0.27)	3.76	<.001	0.23 (0.11 to 0.35)
Mindfulness (MAAS)								
8 wk	566	616	0.39 (0.03)	−0.04 (0.03)	0.43 (0.35 to 0.51)	10.81	<.001	0.63 (0.52 to 0.75)
4 mo	469	562	0.37 (0.03)	−0.08 (0.03)	0.45 (0.38 to 0.53)	11.40	<.001	0.71 (0.59 to 0.84)
BBI, 8 wk								
Total	566	621	−0.34 (0.03)	−0.04 (0.03)	−0.30 (−0.38 to −0.20)	−6.64	<.001	0.39 (0.27 to 0.50)
Exhaustion	576	628	−0.39 (0.04)	−0.08 (0.04)	−0.32 (−0.41 to −0.22)	−6.23	<.001	0.36 (0.25 to 0.47)
Cynicism	576	628	−0.39 (0.04)	−0.08 (0.04)	−0.32 (−0.41 to −0.22)	−6.23	<.001	0.36 (0.25 to 0.47)
Inadequacy	570	623	−0.32 (0.04)	−0.06 (0.04)	−0.26 (−0.37 to −0.14)	−4.46	<.001	0.26 (0.14 to 0.37)
Depression (PHQ-8), 8 wk	577	628	−2.55 (0.17)	−0.27 (0.15)	−2.28 (−2.27 to −1.84)	−10.10	<.001	0.58 (0.47 to 0.70)
Anxiety (GAD-7), 8 wk	574	621	−3.13 (0.19)	−0.42 (0.16)	−2.70 (−3.19 to −2.22)	−10.85	<.001	0.63 (0.51 to 0.75)

Abbreviations: BBI, Bergen Burnout Inventory; GAD-7, 7-item Generalized Anxiety Disorder; MAAS, Mindfulness Attention Awareness Scale; MED, digital meditation intervention; PHQ-8, 8-item Patient Health Questionnaire; WL, waiting list control.

^a^ Independent *t* tests were used to compare means between arms using observed data.

### Secondary Outcomes

#### Job Strain

There was a statistically significant effect of treatment group on changes in effort-reward ratio (Cohen *d*, −0.15; 95% CI, −0.17 to −0.12; *P* < .001) (Table 2 and eFigure 3 in Supplement 1). At 8 weeks, group differences in effort-reward ratio favored MED (Cohen *d*, 0.34; 95% CI, 0.23-0.46; *P* < .001) (Table 3). Those in the MED group showed a mean (SE) reduction of −0.02 (0.00) in Effort-Reward Imbalance Scale scores compared with 0.00 (0.00) in the WL group. At 4 months, group differences continued to favor MED (Cohen *d*, 0.37; 95% CI, 0.25-0.50; *P* < .001). Those in the MED group maintained mean (SE) reduction of −0.02 (0.00) compared with 0.00 (0.00) in the WL group (Table 3). Similarly, there was a statistically significant effect of treatment group on changes in job reward (Cohen *d*, 0.31; 95% CI, 0.20-0.43; *P* < .001) (Table 3) and job effort (Cohen *d*, 0.25; 95% CI, 0.14-0.37; *P* < .001) (Table 3), such that those in the MED group (vs the WL group) showed reductions in effort and increases in reward over time. An exploratory analysis found that increases in 8-week MAAS score explained 23% of the effect of MED (vs WL) on reductions in job strain at 4 months.

#### Work Engagement

There was a statistically significant effect of treatment group on changes in work engagement (Cohen *d*, 0.20; 95% CI, 0.17-0.23; *P* < .001) (Table 2 and eFigure 5 in Supplement 1). At 8 weeks, group differences in total UWES score favored MED (Cohen *d*, 0.31; 95% CI, 0.20-0.43; *P* < .001), as did differences in all 3 subscales (Table 3). Those in the MED group showed a mean (SE) increase of 0.20 (0.03) at 8 weeks compared with 0.01 (0.03) in the WL group. At 4 months, group differences in total UWES score continued to favor MED (Cohen *d*, 0.35; 95% CI, 0.23-0.48; *P* < .001), as did group differences in all 3 subscales (Table 3). At 4 months, participants in the MED group maintained a mean (SE) increase in total UWES score of 0.17 (0.03) compared with a decrease in the WL group (−0.06 [0.03]) (Table 3).

#### Mindfulness

There was a statistically significant effect of treatment group on changes in MAAS (Cohen *d*, 0.38; 95% CI, 0.35-0.41; *P* < .001) (Table 2 and eFigure 7 in Supplement 1). At 8 weeks, the group difference favored MED (Cohen *d*, 0.63; 95% CI, 0.52-0.75; *P* < .001) (Table 3), with those in the MED group showing a mean (SE) increase of 0.39 (0.03), compared with those in the WL group, who showed a mean (SE) reduction of −0.04 (0.03). At 4 months, group differences remained (Cohen *d*, 0.71; 95% CI, 0.59-0.84; *P* < .001) (Table 3), with those in the MED group maintaining mean (SE) increase of 0.37 (0.03) compared with those in the WL group showing a mean (SE) reduction of −0.08 (0.03) (Table 3).

#### Burnout

At 8 weeks, group differences in total BBI score (Cohen *d*, 0.39; 95% CI, 0.27-0.50; *P* < .001) as well as all 3 subscales favored MED (Table 3). Those in the MED group showed a mean (SE) reduction of −0.34 (0.03) compared with −0.04 (0.03) in the WL group (mean difference, −0.30; 95% CI, −0.38 to −0.20; *P* < .001) (Table 3).

#### Depression and Anxiety

At 8 weeks, group difference in PHQ-8 score favored MED (Cohen *d*, 0.58; 95% CI, 0.47-0.70; *P* < .001) (Table 3), with those in the MED group showing a mean (SE) reduction of −2.55 (0.17) compared with −0.27 (0.15) in the WL group (Table 3). At 8 weeks, group difference in GAD-7 score favored MED (Cohen *d*, 0.63; 95% CI, 0.51-0.75; *P* < .001) (Table 3), with those in the MED group showing a mean (SE) reduction of −3.13 (0.19) compared with −0.42 (0.16) in the WL group.

#### Exploratory Analysis of Effect of Treatment Adherence

Among those in the MED group (n = 580 at 8 weeks), the frequency of daily meditation (low, medium, or high) was associated with changes in PSS at 8 weeks (*F*_2,577_ = 4.78; *P* = .009). Those in the high-frequency group (≥10 min/d [30 (5.17%)]: mean score difference, −7.80; 95% CI, −9.69 to −5.91) and medium-frequency group (5-9.9 min/d [146 (25.17%)]: mean score difference, −6.58; 95% CI, −7.44 to −5.73) showed greater reductions compared with those in the low-frequency group (<5 min/d [404 (69.66%)]; mean score difference, −5.42; 95% CI, −5.94 to −4.91). Those in the high-frequency and medium-frequency groups did not statistically differ. These adherence group differences were no longer statistically significant at 4 months.

## Discussion

In this randomized clinical trial, participants randomized to digital meditation showed significant improvements in all study outcomes at 8 weeks after treatment, including reductions in levels of global stress, job strain, work burnout, depression, and anxiety and increases in mindfulness, job reward, and work engagement. The effect size differences ranged from small to large. At the 4-month follow-up, these improvements were maintained. Greater treatment adherence (≥5 min/d of meditation) was associated with greater reductions in perceived stress.

This study confirms prior findings indicating psychological benefits of mindfulness practice for employees^9,10^ and extends them to a digital platform. We found medium to large effect sizes for reductions in PSS and increases in mindfulness across 4 months. The mechanisms by which digital mindfulness interventions impart benefits for both general and work-related stress may include an improved capacity to cope with and positively reappraise stressful situations,^26,27^ as well as enhanced mindfulness^28^ and attention regulation.^29^ In a work context, these enhanced coping abilities may lead to the reappraisal of demands as manageable and work stressors as within one’s control, leading to decreased job strain. In an exploratory analysis, we found that increases in 8-week MAAS score explained 23% of the effect of MED (vs WL) on reductions in PSS and job strain at 4 months.

### Strengths and Limitations

Our study was strengthened by a large sample (>1400) of individuals with moderate to high stress who had no history of meditation practice and by relatively low attrition and low cost. It benefitted from having both work-specific and general measures of stress and well-being as well as an objective measure of treatment adherence through the meditation app. Most participants randomized to digital mindfulness initiated some level of meditation (≥1 min/d) and perhaps meditated more on their own outside the app,^30^ although we were unable to test this directly. Yet, most required at least 1 follow-up contact to improve adherence. Many participants indicated adherence difficulty due to time constraints and technical difficulties. This likely contributed to the lack of a longer-term effect of treatment adherence on study outcomes. Further work should be done to determine the participant experience with digital meditation to enhance adherence (eg, greater outreach, providing incentives, and focus groups to assess burden and overcome barriers).

Our study is limited by a lack of generalizability regarding employee roles, race and ethnicity, and gender. We had a limited sample of participants who identified as service workers, as Black or African American, and as male—groups likely high in work burnout that could potentially benefit the most from our intervention. At the time when this study was conducted, the application was only available in English, likely presenting a barrier to engaging non–English-speaking employees. While attrition was relatively low (16.23%), a greater percentage of attrition occurred among those randomized to MED (19.78%) than among those in the WL group (12.74%). This was perhaps due to perceived treatment burden. We were also unable to ascertain whether treatment effects were due to nonspecific factors (eg, time, attention) or specific mindfulness-based and digital-specific factors.

## Conclusions

In this randomized clinical trial, a digital mindfulness meditation intervention reduced perceptions of stress, job strain, and burnout and improved work engagement, mindfulness, and general well-being among employees at a large academic medical center. As we transition to a “new normal” in a postpandemic world, there is an urgent need to identify scalable solutions to improve employee well-being. Given the strong effects of digital mindfulness and the low cost, greater dissemination across workplace settings could be beneficial. Future studies are needed to enhance adherence and to better characterize treatment mechanisms. Workplaces may also consider making system-level changes to enhance employee well-being. Given well-established links between chronic work stress and metabolic dysregulation^3,4,5,6,7^ and that our group’s pilot study found decreases in waist circumference with this same intervention,^31^ future research should also investigate downstream health benefits and employer health savings.

## Notes

Supplement 1.
Trial Protocol and Statistical Analysis Plan

Supplement 2. **eTable 1.** Attrition Analysis Due to Dropout for Primary and Secondary Outcomes **eTable 2.** Sensitivity Analysis for Mixed Models With and Without Cutoff Thresholds **eFigure 1.** Grouped Line Plots for Perceived Stress Scale Scores **eFigure 2.** Quality Control Plots for the Linear Mixed-Effect Model Using Perceived Stress Scale Scores **eFigure 3.** Grouped Line Plots for Effort-to-Reward Ratios **eFigure 4**. Quality Control Plots for the Linear Mixed-Effect Model Using Effort-to-Reward Ratios **eFigure 5.** Grouped Line Plots for Work Engagement Scores **eFigure 6.** Quality Control Plots for the Linear Mixed-Effect Model Using Work Engagement Scores **eFigure 7**. Grouped Line Plots for Mindfulness Attention Awareness Scores **eFigure 8.** Quality Control Plots for the Linear Mixed-Effect Model Using Mindfulness Attention Awareness Scores

Supplement 3.
Data Sharing Statement
