# Supplementary material for: Digital Meditation to Target Employee Stress: A Randomized Clinical Trial
Source: JAMA Netw Open. 2025 Jan 14;8(1):e2454435. doi: 10.1001/jamanetworkopen.2024.54435 (PMC11733700; doi:10.1001/jamanetworkopen.2024.54435)
Supplement: Supplement 2. — eTable 1. Attrition Analysis Due to Dropout for Primary and Secondary Outcomes eTable 2. Sensitivity Analysis for Mixed Models With and Without Cutoff Thresholds eFigure 1. Grouped Line Plots for Perceived Stress Scale Scores eFigure 2. Quality Control Plots for the Linear Mixed-Effect Model Using Perceived Stress Scale Scores eFigure 3. Grouped Line Plots for Effort-to-Reward Ratios eFigure 4. Quality Control Plots for the Linear Mixed-Effect Model Using Effort-to-Reward Ratios eFigure 5. Grouped Line Plots for Work Engagement Scores eFigure 6. Quality Control Plots for the Linear Mixed-Effect Model Using Work Engagement Scores eFigure 7. Grouped Line Plots for Mindfulness Attention Awareness Scores eFigure 8. Quality Control Plots for the Linear Mixed-Effect Model Using Mindfulness Attention Awareness Scores [file jamanetwopen-e2454435-s002.pdf]

Supplementary Online Content

Radin RM, Vacarro J, Fromer E, et al. Digital meditation to target employee stress: a randomized clinical trial. *JAMA Netw Open*. 2025;8(1):e2454435. doi:10.1001/jamanetworkopen.2024.54435

eTable 1. Attrition Analysis Due to Dropout for Primary and Secondary Outcomes
eTable 2. Sensitivity Analysis for Mixed Models With and Without Cutoff Thresholds
eFigure 1. Grouped Line Plots for Perceived Stress Scale Scores
eFigure 2. Quality Control Plots for The Linear Mixed-Effect Model Using Perceived Stress Scale Scores
eFigure 3. Grouped Line Plots for Effort-to-Reward Ratios
eFigure 4. Quality Control Plots for the Linear Mixed-Effect Model Using Effort-to-Reward Ratios
eFigure 5. Grouped Line Plots for Work Engagement Scores
eFigure 6. Quality Control Plots for the Linear Mixed-Effect Model Using Work Engagement Scores
eFigure 7. Grouped Line Plots for Mindfulness Attention Awareness Scores
eFigure 8. Quality Control Plots for the Linear Mixed-Effect Model Using Mindfulness Attention Awareness Scores

This supplementary material has been provided by the authors to give readers additional information about their work.

© 2025 Radin RM et al. *JAMA Network Open*.

eTable 1. Attrition analysis due to drop out for primary and secondary outcomes.

**Variable**	**Follow-up**	***N* MED**	***N* WL**	**Mean (SD) baseline value for drop-outs (MED)**	**Mean (SD) baseline value for drop-outs (WL)**	***p*-value**
PSS	8wk	580	634	21.25 (4.90)	20.96 (5.27)	0.66
	4mo	489	585	21.42 (5.14)	20.98 (4.83)	0.55
MAAS	8wk	566	616	3.37 (0.85)	3.44 (0.93)	0.99
	4mo	469	562	3.37 (0.84)	3.49 (0.98)_	0.67
Job Strain - Reward	8wk	567	625	0.22 (0.06)	0.23 (0.06)	0.72
	4mo	473	571	0.23 (0.07)	0.22 (0.06)	0.48
Job Strain -Effort	8wk	578	625	3.52 (0.82)	3.49 (0.91)	0.79
	4mo	475	571	3.52 (0.92)	3.44 (0.97)	0.43
BBI average	8wk	566	621	3.20 (0.97)	3.23 (0.99)	0.79
PHQ	8wk	577	628	8.19 (4.86)	7.28 (4.76)	0.14
Anxiety	8wk	574	621	8.43 (4.78)	8.33 (5.18)	0.88

© 2025 Radin RM et al. *JAMA Network Open*.

eTable 2. Sensitivity analysis for linear mixed models with and without cutoff thresholds.

Variable	Mean (CI) with thresholds	Mean (CI) without thresholds	p-value
PSS	-2.55 [-2.91, -2.20]	-2.55 [-2.90, -2.20]	0.91
Job Strain	-0.0096 [-0.0138, -0.0053]	-0.0096 [-0.0138, -0.0053]	0.99
Work Engagement	0.18 [0.12, 0.24]	0.18 [0.12, 0.24]	0.92
MAAS	0.31 [0.25, 0.36]	0.31 [0.26, 0.36]	0.89

© 2025 Radin RM et al. *JAMA Network Open*.

eFigure 1. Grouped line plots for Perceived Stress Scale (PSS) scores are provided at Baseline, 8 weeks, and 4 months post-randomization.

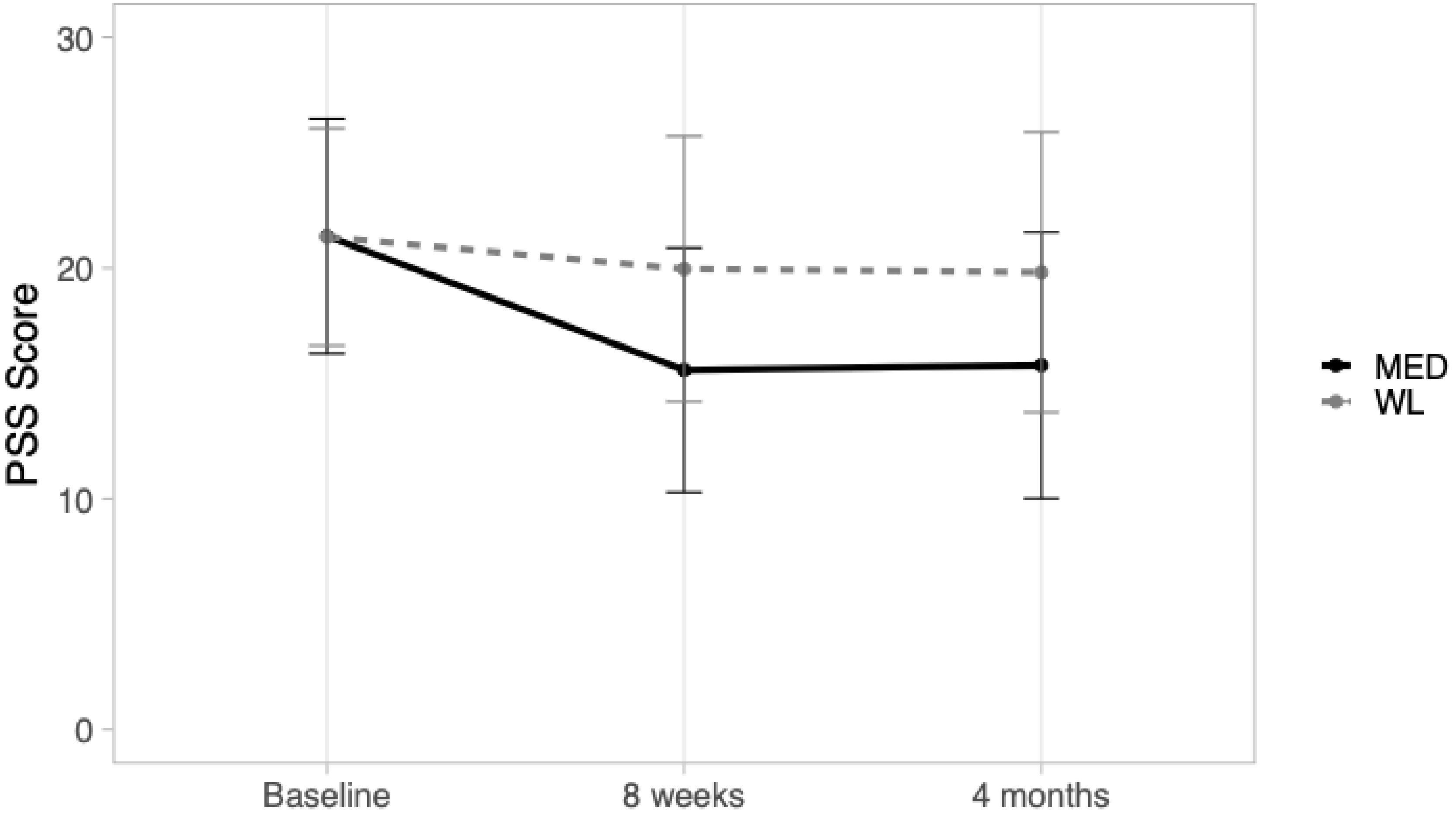

© 2025 Radin RM et al. *JAMA Network Open*.

eFigure 2. Quality control plots for the linear mixed-effect model using Perceived Stress Scale scores. Posterior predictions (top-left), linearity (top-right), normality of the residuals (bottom-left), and normality of the random effects (bottom-right) were tested to confirm model assumptions and gauge model performance.

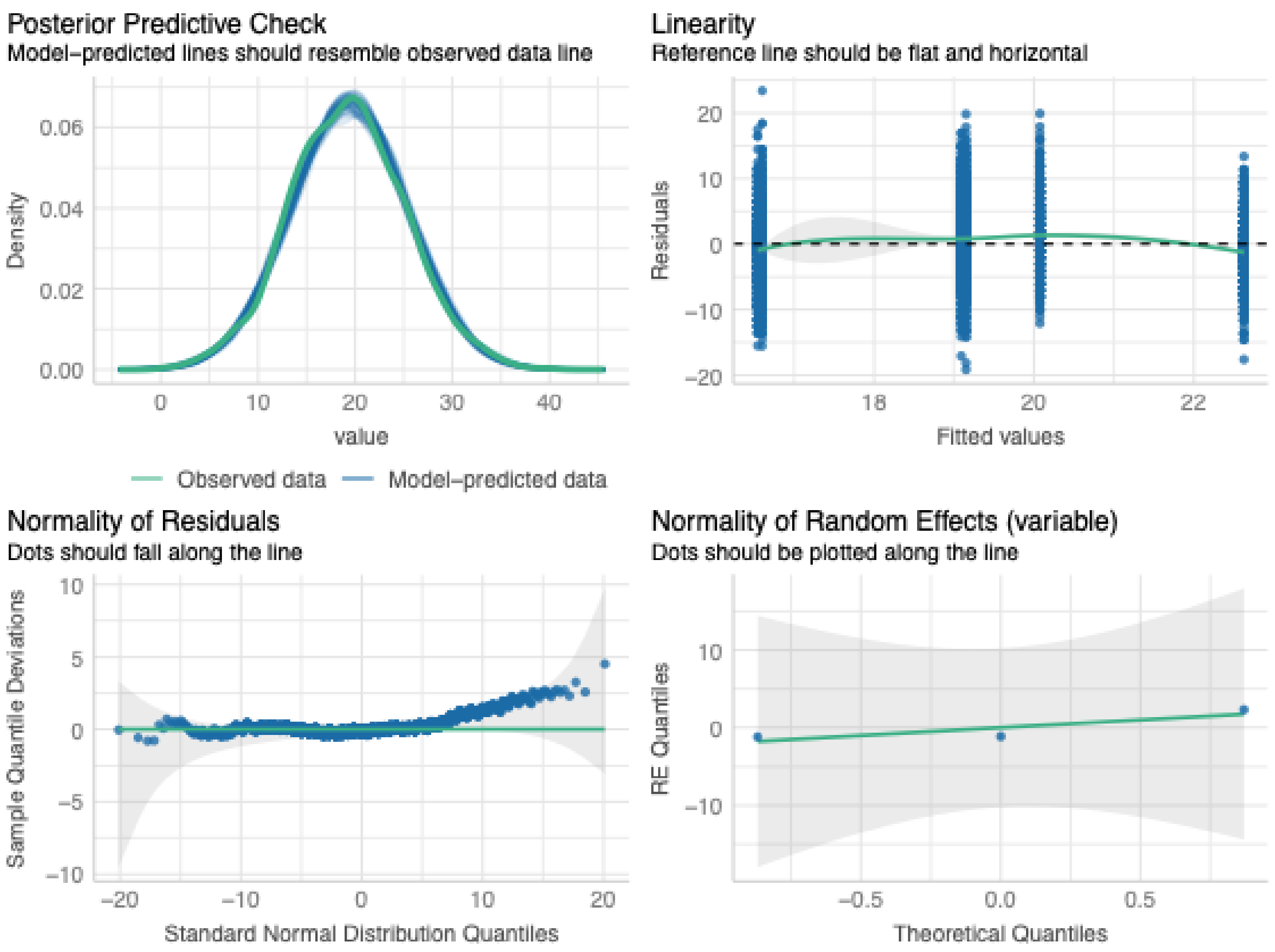

© 2025 Radin RM et al. *JAMA Network Open*.

eFigure 3. Grouped line plots for Effort-to-reward (ER) ratios are provided at baseline (BL), 8 weeks (T1), and 4 months (T2) post-randomization. Two-sided p-values were calculated for each censoring time using ANOVA.

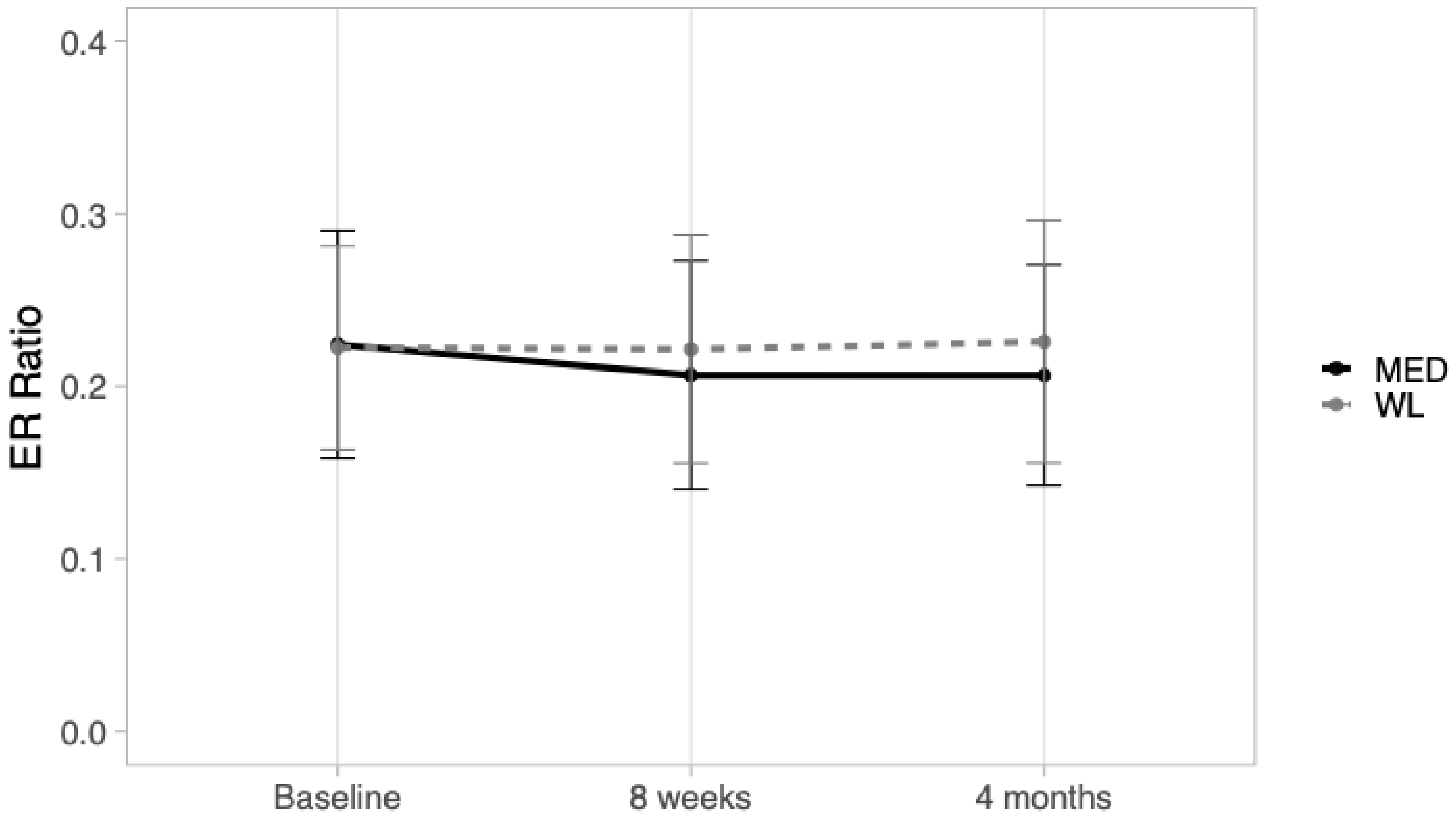

© 2025 Radin RM et al. *JAMA Network Open*.

eFigure 4. Quality control plots for the linear mixed-effect model using Effort-to-reward ratios. Posterior predictions (top-left), linearity (top-right), normality of the residuals (bottom-left), and normality of the random effects (bottom-right) were tested to confirm model assumptions and gauge model performance.

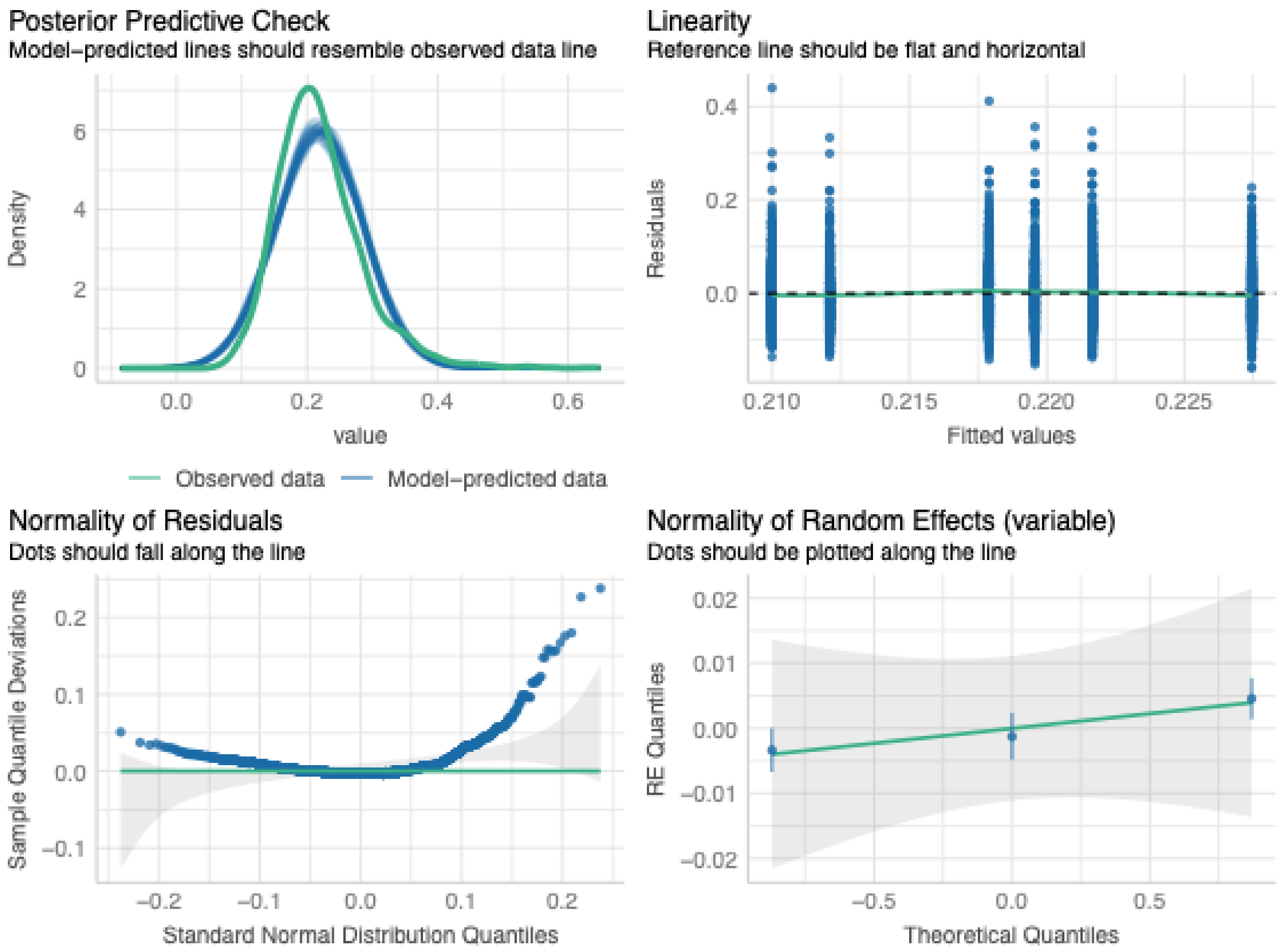

© 2025 Radin RM et al. *JAMA Network Open*.

eFigure 5. Grouped line plots for Work Engagement scores (UWES) are provided at baseline (BL), 8 weeks (T1), and 4 months (T2) post-randomization. Two-sided p-values were calculated for each censoring time using ANOVA.

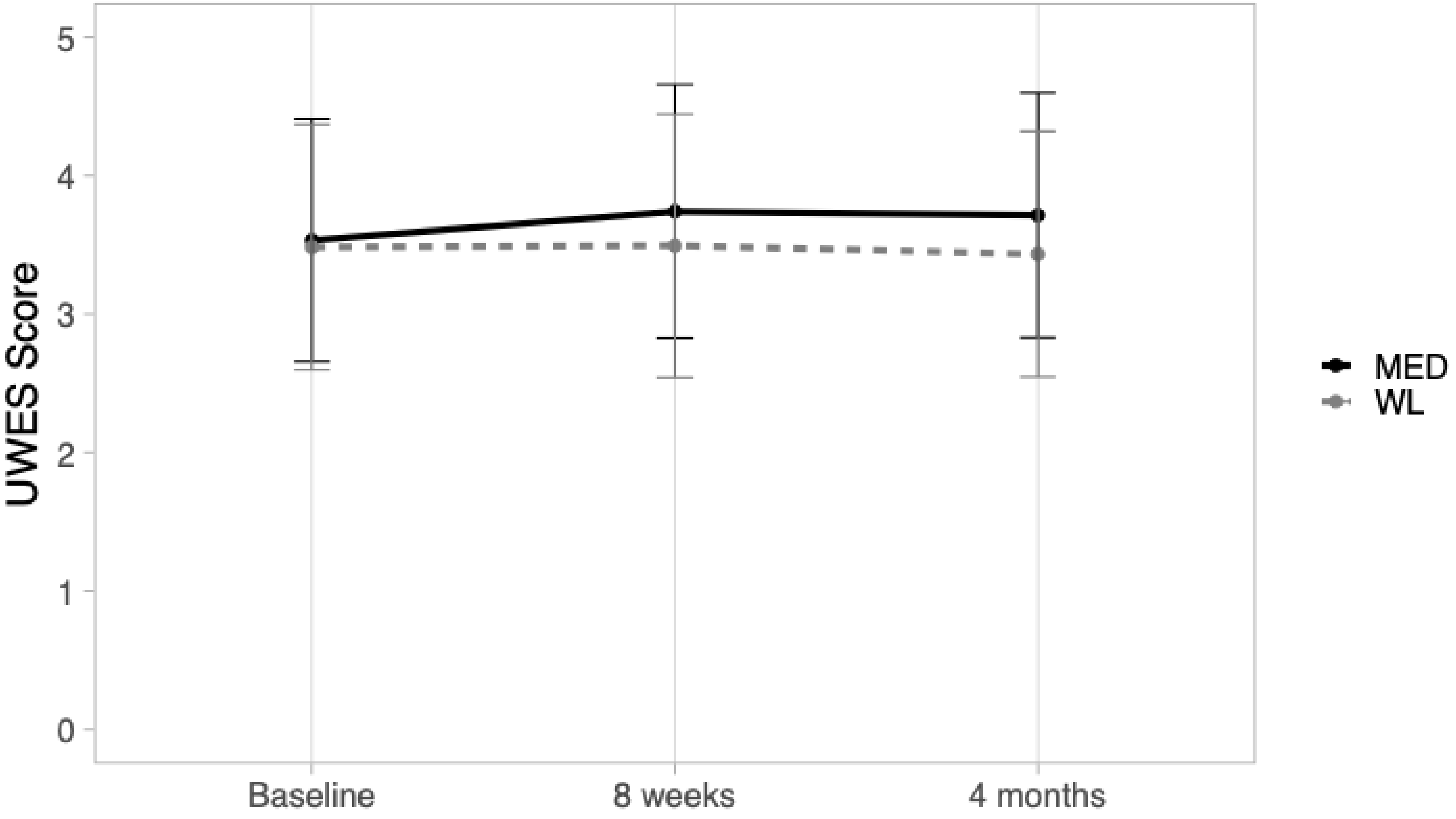

© 2025 Radin RM et al. *JAMA Network Open*.

eFigure 6. Quality control plots for the linear mixed-effect model using Work Engagement scores. Posterior predictions (top-left), linearity (top-right), normality of the residuals (bottom-left), and normality of the random effects (bottom-right) were tested to confirm model assumptions and gauge model performance.

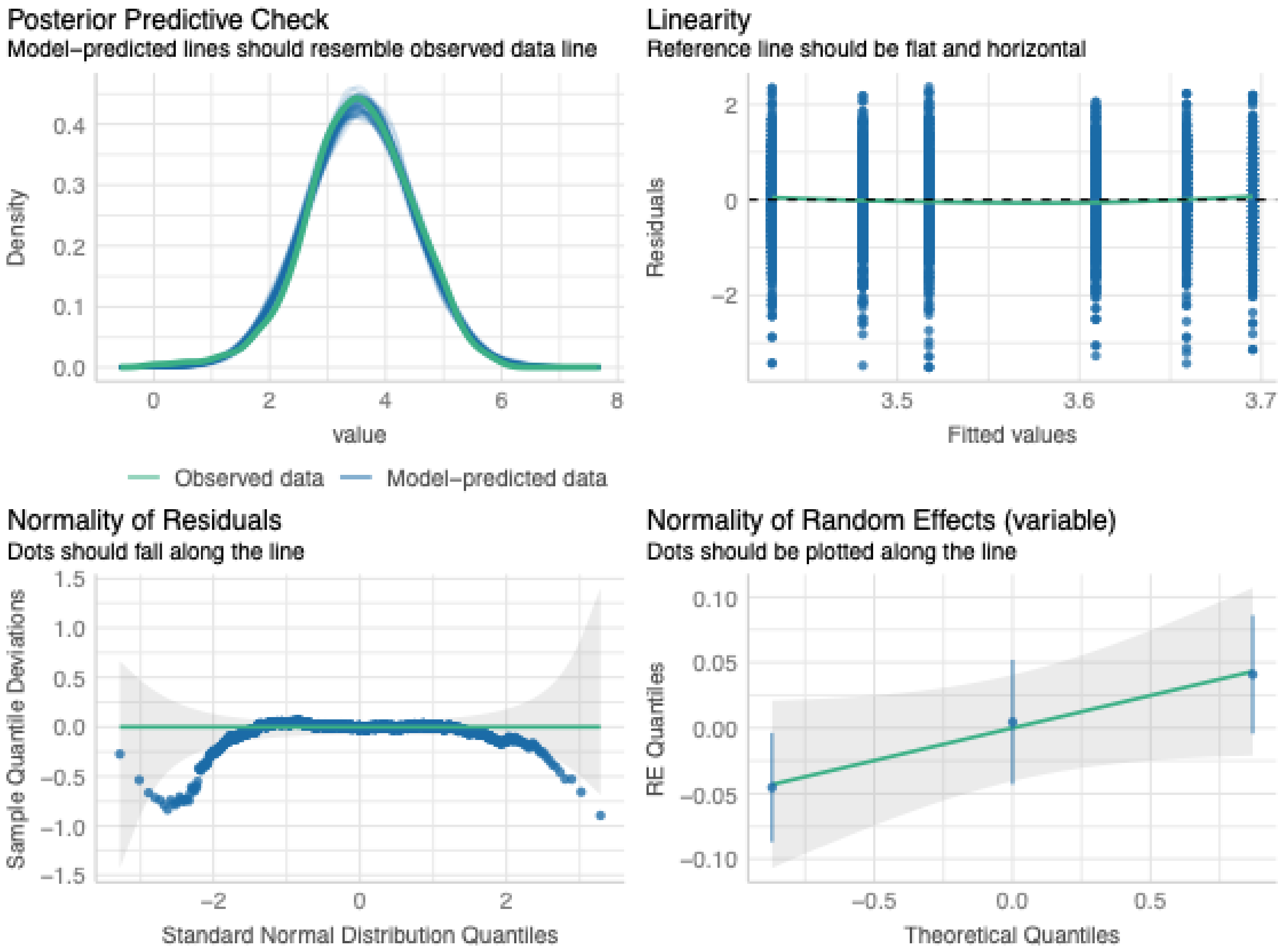

© 2025 Radin RM et al. *JAMA Network Open*.

eFigure 7. Grouped line plots for Mindfulness Attention Awareness Scale (MAAS) scores are provided at baseline (BL), 8 weeks (T1), and 4 months (T2) post-randomization. Two-sided p-values were calculated for each censoring time using ANOVA.

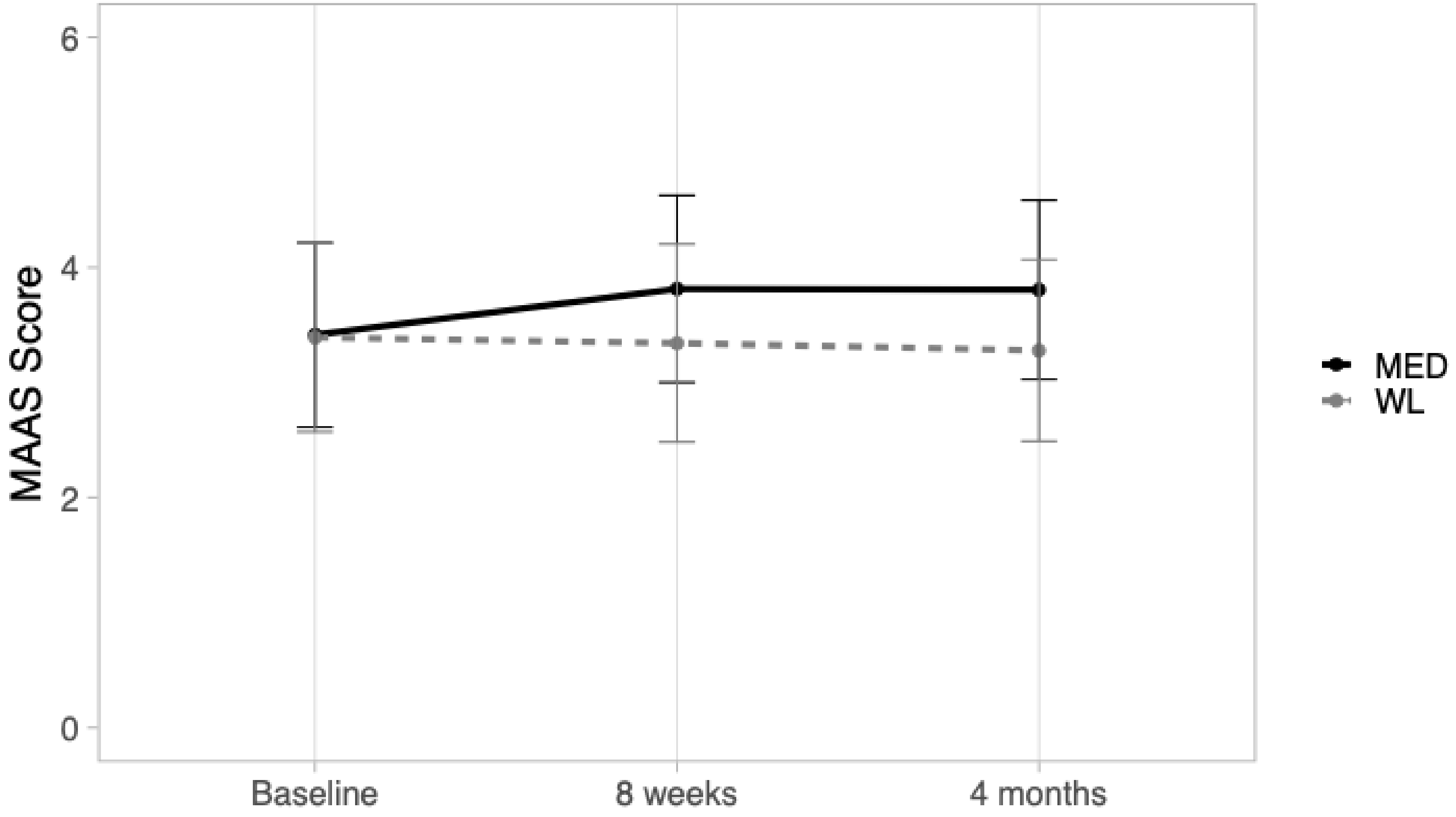

© 2025 Radin RM et al. *JAMA Network Open*.

eFigure 8. Quality control plots for the linear mixed-effect model using Mindfulness Attention Awareness Scale scores. Posterior predictions (top-left), linearity (top-right), normality of the residuals (bottom-left), and normality of the random effects (bottom-right) were tested to confirm model assumptions and gauge model performance.

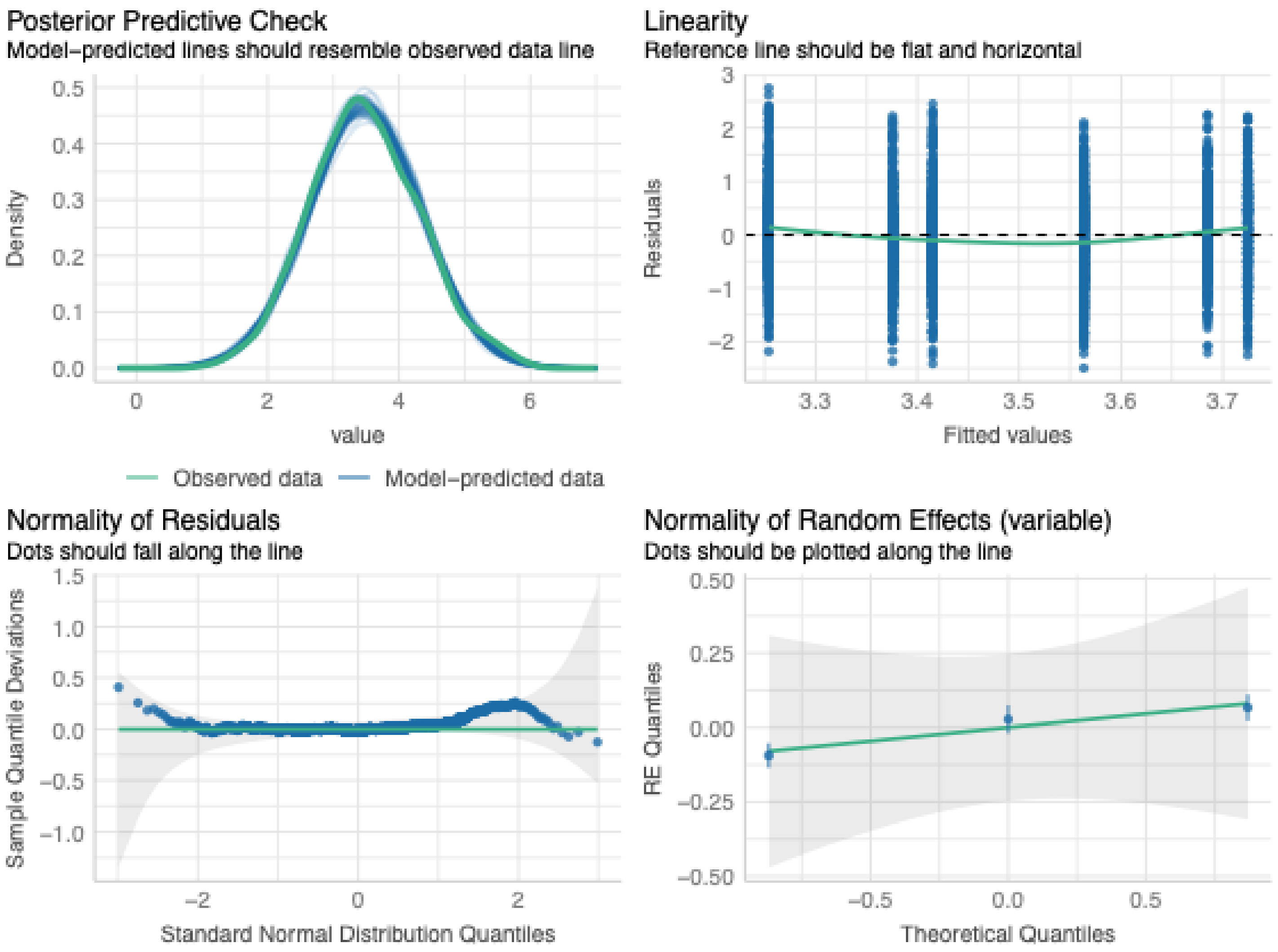

© 2025 Radin RM et al. *JAMA Network Open*.
