# Supplementary material for: Digital Meditation to Target Employee Stress: A Randomized Clinical Trial
Source: JAMA Netw Open. 2025 Jan 14;8(1):e2454435. doi: 10.1001/jamanetworkopen.2024.54435 (PMC11733700; doi:10.1001/jamanetworkopen.2024.54435)
Supplement: Supplement 3. — Data Sharing Statement [file jamanetwopen-e2454435-s003.pdf]

Data Sharing Statement

Radin. Digital Meditation to Target Employee Stress. *JAMA Netw Open*. Published January 14, 2025. doi:10.1001/jamanetworkopen.2024.54435

Data
Additional Information: clinicaltrials.gov, NCT03527303, https://clinicaltrials.gov/study/NCT03527303
Data available: Yes
Data types: Deidentified participant data
How to access data: Data will be made available on Open Science Framework (OSF) upon manuscript acceptance.
When available: With publication

Supporting Documents
Document types: Statistical/analytic code
How to access documents: Data will be made available on Open Science Framework (OSF) upon manuscript acceptance.
When available: With publication

Additional Information
Who can access the data: Anyone requesting the data
Types of analyses: For any purpose
Mechanisms of data availability: With a signed data access agreement
